# Accuracy of a Self-monitoring Test for Identification and Monitoring of Age-related Macular Degeneration: A Diagnostic Case-control Study

**DOI:** 10.2174/1874364101812010019

**Published:** 2018-03-13

**Authors:** Martin K. Schmid, Livia Faes, Lucas M. Bachmann, Michael A. Thiel

**Affiliations:** 1 Eye Clinic, Cantonal Hospital of Lucerne, Lucerne, Switzerland; 2 Medignition Inc, Research Consultants, Zurich, Switzerland

**Keywords:** Age-dependent macular degeneration, Screening, Diagnosis, Sensitivity, Specificity, Self-monitoring, Diagnostic case-control study

## Abstract

**Objective:**

To evaluate diagnostic accuracy of a new self-monitoring device using a Vernier hyperacuity alignment task.

**Method:**

A total of 11 wet Age-Related Macular Degeneration (AMD) patients and 9 controls contributing 37 eyes were consecutively enrolled into this prospective diagnostic case-control study at the retina centre of the Cantonal Hospital Lucerne, Switzerland. Vernier acuity testing (index test) and Optical Coherence Tomography (OCT, reference test) were performed in all participants. OCT scans were evaluated and graded by a retinal specialist masked to diagnosis and index test results. Candidate parameters of the index test to be used as the diagnostic statistic were identified using a bootstrap procedure. Ten parameters remaining were further assessed in univariate analyses. The overall Standard Deviation (SD) of absolute distances across all four axes of the Vernier acuity test provided the highest area under the Receiver Operating Characteristics (ROC) curve and was therefore selected.

**Results:**

Mean age of patients with wet AMD was 81.2 years (SD 4.99), mean numbers of letters were 67.4 (SD 14.1). The proportion of women was similar in both groups (controls: 88%, wet AMD: 72%). The area under the ROC curve was 0.87 (95% confidence interval CI: 0.75- 0.99) indicating excellent discrimination. Best accuracy was reached at a cut-off value of 0.64 with a sensitivity of 75% and a specificity of 94%.

**Conclusion:**

This diagnostic case-control study of a new screening device for AMD shows acceptable diagnostic accuracy. The promising preliminary data of this study call for further upstream evaluations in reasonably sized clinical studies.

## 1 INTRODUCTION

The availability of highly effective new treatment modalities in wet AMD have re-fuelled the discussion about the importance of early identification and targeted patient monitoring [1, 2]. Ideally, this could be achieved with a mobile screening and monitoring device that is easy to handle and also valid in the hands of a patient. However, to date, the Amsler grid is still one of the most frequently used and recommended self-monitoring tests in clinical practice. In their study, Crossland and Rubin found large variability of its diagnostic accuracy and discussed various obstacles such as difficulty with fixation in self-monitoring experiments [3]. In view of the fact that the majority of patients with suspected AMD already have some degree of visual impairment, regular physician visits are impractical. The lack of a simple test that could be performed at home may lead to unnecessary delay of diagnosis, in the extreme case at a stage, where permanent vision loss due to retinal damage has already occurred.

We are aware of several technologies used for self-monitoring in AMD such as *i.e*. the Preferential Hyperacuity Perimeter (PHP) [4]. One recent trial showed an advantage of PHP home-monitoring (ForeseeHome^®^, Notal Vision Ltd, Tel Aviv, Israel) in patients with Choroidal Neovascularisations (CNV), because regular home measurements discovered new CNV onset at an earlier stage [5]. However, some authors pointed at the disadvantages particularly in terms of user-friendliness and complexity of the task [6, 7].

In this study, we describe a new self-monitoring test based on a Vernier hyperacuity alignment task. Within the context of a diagnostic case-control study, we evaluated its diagnostic accuracy for the screening and monitoring of patients with AMD.

## 2 MATERIAL AND METHODS

The local Ethics Committee approved this study.

### 2.1 Study Design and Setting

This study was designed as a prospective, clinical study with consecutive patient enrolment. All patients attending an ophthalmological consultation between June and August 2013 at the Retina Centre of the Cantonal Hospital of Lucerne’s Eye Clinic were screened for inclusion. Healthy controls were a sample of consenting eligible staff members of the eye clinic.

### 2.2 Patient Recruitment and Enrolment

Patients with wet AMD in a pro re-nata regimen were approached by the treating ophthalmologist who checked the inclusion criteria. In the positive case, he or she provided detailed oral and written information about the study and asked whether the patient was willing to participate. Participating patients and healthy controls provided written informed consent.

### 2.3 Inclusion and Exclusion Criteria

To be included in this study, patients needed to have cases of active CNV secondary to AMD, either newly diagnosed (treatment-naïve) or treated with anti–VEGF agents (ranibizumab (Lucentis ^®^) or aflibercept (Eylea ^®^)).

We excluded patients with a neurological or physical illness that impeded from performing the test adequately.

### 2.4 Examination Setting

Salient clinical characteristics of each patient were secured. The screening test was then conducted between the ophthalmological examination and the intravitreal injection. After receiving a careful instruction on how to perform the test using a standardised protocol, each patient performed the test on its own. If needed, the study coordinator provided technical assistance. The necessity to do so was registered. All participants performed the test wearing their usual corrected spectacles. Participants with progressive bifocals or multi-focal glasses performed the test with adapted glasses. This was a monocular test. Eyes were tested separately.

### 2.5 Index Test: Vernier Acuity Testing

The test is based on a computerized version of a Vernier hyperacuity alignment task. Vernier acuity is the ability to detect a misalignment among lines or dots and is more accurate than visual acuity. The Vernier acuity is deteriorated by morphological retinal changes that cause metamorphopsia. Therefore, measurement of Vernier acuity can provide information of retinal changes. Moreover, Vernier acuity testing is less influenced by surrounding conditions than visual acuity testing and hence best suited for self-testing.

Participants (*i.e.* patients and healthy controls) were seated approximately 30 centimeters from the monitor. Three white dots having a diameter of one millimeter on black screen were presented, either aligned vertically, horizontally or in left as well as right oblique direction Fig. (**1**)

Patients had to align the three randomly misaligned dots exactly on one axis by only moving the white dot in the middle. Patients were able to control the middle dot by the four arrows of a keyboard and confirmed the final position pressing the space bar. This task was repeated ten times for each of the four axes. For each participant, the program secured the distance of the middle point to the corresponding axis resulting in a total of 40 inputs per eye.

### 2.6 Reference Testing

All study participants underwent OCT testing to confirm the presence and extent of wet AMD. A spectral domain OCT (Spectralis® Heidelberg Engineering GmbH, Heidelberg) was used to obtain four crossline scans with a length of six millimetres each. OCT used the same four axes that were used for the Vernier acuity test. An experienced retinal specialist from the eye clinic in Lucerne graded and classified morphological changes of the retina based on the OCT scans. The grading of the OCT scans was done independently and without knowing the corresponding result of the index test and considered the inner retinal surface, the photoreceptor layers and the pigment epithelium. Normal morphology had a score value of zero. Each subscore had four levels, thus the total score could have values between zero and 12 points and was based on the morphological appearance of each retinal layer.

### 2.7 Statistical Analysis

#### 2.7.1 Descriptive Statistics

Continuous variates were summarised with means and SD of medians and Interquartile Ranges (IQR) if data were skewed. Dichotomous variates were described as rates and percentages.

For each eye, we calculated the absolute overall distance and the corresponding SD per axis and the overall absolute distance and SD across all axes.

Statistical comparison of groups of eyes was done using non-parametric methods.

#### 2.7.2 Modeling

In univariate and multivariate analyses we examined which (set of) parameters (independent variates) provided the best possible discrimination between AMD patients and controls (dependent variate). We bootstrapped a stepwise forward regression model 100 times and counted the number of times each variate was selected in the final model. Parameters remaining in the final model in at least 80 percent of bootstrapping cycles were further examined. For each of the remaining parameters we fitted a ROC curve for diagnosis of AMD by each of the test parameters, using a maximum likelihood logistic regression model based on robust standard errors. The area under the receiver operating characteristics curve (AUC) and its corresponding 95% CI was estimated. In analogy to Kappa values, we interpreted AUC values as proposed by Landis and Koch [8]. Highest sensitivity values at 100 percent specificity and vice versa were determined.

The overall SD of absolute distances across all four axes of the Vernier acuity test provided the highest AUC and was therefore selected. The final model was a mixed linear model using subject as a random factor. This model adjusted for the fact that measurements were independent between subjects but dependent within subjects if a subject provided data on both eyes. Analyses were done using the Stata 11.2 statistical software package (StataCorp. 2009. Stata Statistical Software: Release 11. College Station, TX: StataCorp LP.).

## 3 RESULT

### 3.1 Patient’s Descriptive

**Fig. (2):**
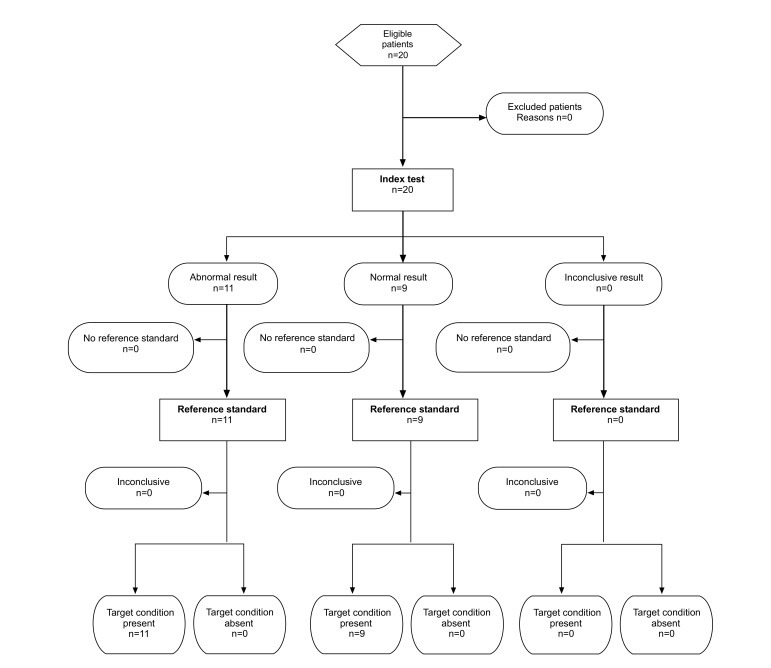
STARD flow diagram of 20 study participants (11 patients with AMD and 9 healthy controls) contributing with a total of 37 eyes.

A total of 20 participants (11 with AMD and 9 healthy controls) contributing with 37 eyes were enrolled between June and August 2013. All subjects with the exception of three contributed with both eyes to the analysis. In those three cases, a low visual acuity of the second eye did not permit to complete the test. However, we were able to include all the results of participants that fully completed the test, no results had to be excluded in terms of lacking reliability (Fig. **2**).

Control participants (mean (SD): 31.2 years (8.4)) were younger on average than patients suffering from wet AMD (mean (SD): 81.2 years (4.99)). The proportion of women was similar in both groups (controls: 88%, wet AMD: 72%). The median numbers of letters in patients with wet AMD were 71.5 (IQR 62 to 71).

### 3.2 Index Test: Vernier Acuity Testing

The mean test time for Vernier acuity testing -not including patient’s instructions, provision of refractive correction, positioning of the patient and saving of the results was 470 (SD 298) seconds. Patients with wet AMD needed 596 (SD 338) seconds to complete the test and healthy subjects 313 (SD 120) seconds (*p*=0.003). On average, participants needed more time for the first eye tested than for the second presumably because of a training effect. Patients with wet AMD needed 762 (SD 361) seconds for the first eye and 393 (SD 157) for the second eye (*p*=0.01). Healthy subjects needed 389 (SD 83) seconds for the first eye and 216 (SD 84) for the second eye (*p*=0.001).

### 3.3 Problems During Testing

Several patients needed assistance with the handling of the computer (*i.e.* the positioning of the fingers on the arrows of the keyboard), some needed repetitive instructions about the task und one patient needed a short break while completing the test due to concentration problems. Additionally, some patients had to be reminded to remain in the working distance of about 30 centimeters to the monitor. Some patients tried to change the positioning of the head searching for a preferred retinal locus to observe the target. No adverse events occurred while performing the test.

Fig. (**3**) shows the association between test result and OCT score values. (6.32 (95% CI: 3.68 to 8.96; *p*<0.001).

### 3.4 Distribution of Parameters

Table (**1a**) shows the distribution of parameters of the index test for all four axes by classification of the reference test.

### 3.5 Reference Testing

Table (**1b**) shows the grading of OCT scans of all study subjects scoring morphological characteristics of the inner retinal surface, the retinal structure, the photoreceptor layers and the pigment epithelium. The average score for inner retinal surface was 1.25, for retinal structure 1.1, for photoreceptor layers 1.8 and for pigment epithelium 2.05. The average total score was 6.2. The OCT as standard reference testing for wet AMD was conducted subsequently to the Vernier acuity testing without administering any treatment in between. No adverse events occurred administering OCT as standard reference testing.

### 3.6 Test Accuracy of the Diagnostic Algorithm

The ROC curve is shown in Fig. (**4**). The corresponding AUC was 0.87 (95%CI: 0.75 to 0.99) indicating excellent discrimination. At the cut-off value for the test parameter of 0.82, specificity reached 100 percent at a sensitivity of 65 percent. Correspondingly, at sensitivity of 100 percent, specificity was as low as 6 percent. Best accuracy (maximal Youden Index (J)) was reached at a cut-off value of 0.64 with a sensitivity of 75 percent and a specificity of 94 percent.

## 4 DISCUSSION

### 4.1 Main Findings

In this diagnostic case-control study of limited size, a simple diagnostic algorithm based on the variability of aberrations in a visual task implemented in a new self-screening device for the onset and progression of AMD was highly accurate. We also found a high concordance between the values of the diagnostic parameter and the severity of AMD as quantified on a multidimensional scale. The test was easy to perform and all study participants were able to complete the task without difficulty. This test, assessing the Vernier acuity appears to be a promising tool in the management of patients with new onset of AMD or under treatment with anti-VEGF agents.

### 4.2 Results in Context of the Existing Literature

Due to the re-fuelling of the discussion about early detection and sensitive monitoring of wet AMD by the availability of new treatment options, it would be useful having a cost effective, transportable and easily feasible diagnostic tool with high accuracy. Crossland and Rubin already proclaimed the need of mobile screening devices in 2007 and Loewenstein and co-workers showed in a randomised controlled trial that visual acuity was better preserved using a home monitoring system compared to standard care [3, 5]. Kaiser and co-workers showed that the generation of elderly people to-date is already capable, willing and compliant to use technological tools with appropriate guidance and instructions [6]. Currently, we are only aware of several self-monitoring technologies, *i.e.* one developed by Loewenstein based on PHP [4] (ForeseeHome^®^), one by Chhetri *et al.* [9] used in a health management tool by Kaiser *et al.* (myVisiontrack^®^) [6]. Table (**2**), shows sensitivity and specificity values of self-monitoring technologies in AMD reported in a recent systematic review of our group that was published in 2014 [10]. Compared to the PHP, for which diagnostic accuracy has been assessed most comprehensively, our findings are well within boundaries (values of sensitivity between 68 percent [11] and 100 percent [12] and specificities between 71 percent [11] and 97 percent [13] have been reported).

### 4.3 Strength and Limitations

Our study was conducted according to up-to-date guidelines for diagnostic accuracy studies and avoided typical problems of early evaluation studies such as partial verification, unmasked assessment of reference and index test and other methodological aspects introducing bias [14, 15]. This was the first study exploring the potential of this test based on the Vernier acuity for the screening and monitoring of wet AMD. Given the exploratory nature of this study, we did not perform a formal sample size analysis [16]. Due to the small study size we were unable to study subgroups of AMD severity and other stratified analyses. Moreover, we used a so called diagnostic case-control design, which usually exaggerates test performance characteristics [14, 15, 17]. While this approach seems to be rational in early evaluations of tests, their results should not be extrapolated mindlessly into clinical practice.

### 4.4 Implications for Research

Given the encouraging first results of this novel test, further upstream evaluations seem justified [18]. First and foremost, evaluations in a reasonably sized prospective cohort study enrolling patients with suspected AMD should be performed. If test performance remains at acceptable levels, usefulness in clinical practice needs to be assessed. In particular, the value in the (mass) screening setting and as a monitoring tool, *i.e.* the optimization of treatment outcomes when performing home measurements needs to be investigated.

### 4.5 Implications for Practice

In our view, no direct clinical implications emerge from this study. All depends on the extent to which our preliminary findings translate into more comprehensive assessments. However, if confirmed, our new test could have substantial impact on patient care – both in the way we identify patients at risk and individualize medical care. Due to the relatively simple setup of this test, it could be easily programmed into smartphones and other mobile devices. We are aware of only one such tool currently and consider this to be the way forward [19]. Adopting the role model of patient centred diabetes care [20], we believe that involving patients in the early detection and management of AMD will have a major impact on the protection of sight.

## CONCLUSION

This first diagnostic case-control study of a new screening device for AMD shows acceptable diagnostic accuracy. The promising preliminary data found in this study call for further upstream evaluations in reasonably sized clinical studies.

## Figures and Tables

**Fig. (1):**
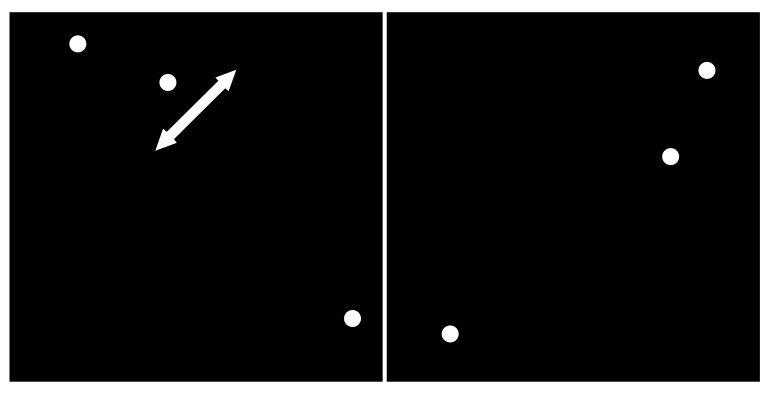
Examples of tasks. The white arrow indicates the directions for shifting the middle white dot between the two outer dots. The task is to set the middle point in a straight line to the outer two dots.

**Fig. (3):**
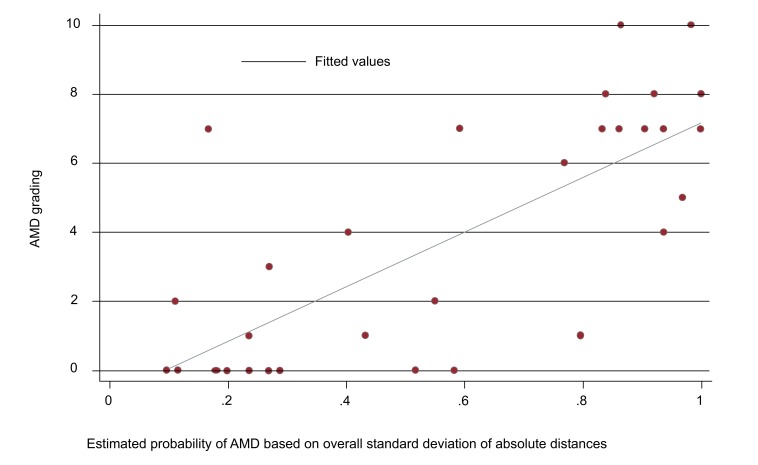
The correlation between estimated probabilities from the diagnostic model and the corresponding AMD grading based on the OCT.

**Fig. (4):**
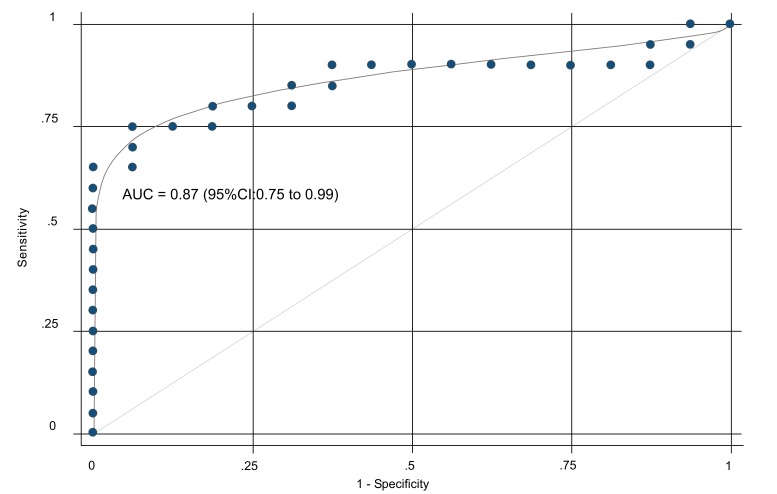
shows the ROC curve with associated AUC.

**Table 1a:** Distribution of parameters of the index test for all four axes by grading of the reference test of 37 eyes from 20 subjects.

**ID**	**Axis 1**	**Axis 2**	**Axis 3**	**Axis 4**	Grade OCT*
1	*6.8*	*9.3*	*6.9*	*9.9*	*5*
2	*5.9*	*3.9*	*13.5*	*9.6*	*10*
3	*7*	*6.2*	*7.3*	*9.8*	*8*
4	*4.2*	*11.7*	*2.3*	*11.4*	*5*
5	*1.6*	*4.6*	*4.1*	*3*	*3*
6	*7.6*	*20.7*	*12.9*	*9.4*	*7*
7	*2*	*3.8*	*2.4*	*2.8*	*7*
8	*1.1*	*3.5*	*0.9*	*1.9*	*2*
9	*6.4*	*8.8*	*3.8*	*7.9*	*7*
10	*1.8*	*5.5*	*3.6*	*9*	*7*
11	*4.6*	*9.9*	*4.7*	*10.6*	*7*
12	*16.8*	*16.4*	*11.5*	*17.9*	*8*
13	*7.1*	*6.1*	*5.4*	*5.6*	*8*
14	*7.7*	*5.1*	*4.7*	*4.6*	*2*
15	*3.5*	*6.7*	*10*	*6.5*	*10*
16	*2.7*	*12.8*	*9.1*	*7.2*	*7*
17	*3.9*	*16.1*	*3*	*3.6*	*4*
18	*2*	*9.1*	*2.6*	*3*	*4*
19	*1.9*	*11.1*	*2.8*	*6.4*	*6*
20	*3.8*	*12.2*	*1.2*	*4.7*	*7*
21	*1.2*	*1.9*	*2.2*	*3.2*	*0*
22	*1.3*	*2.4*	*1.4*	*3*	*0*
23	*1.6*	*5.3*	*2.8*	*6.1*	*0*
24	*2.6*	*3.6*	*1.5*	*4.6*	*0*
25	*2.6*	*5.2*	*3.5*	*7.5*	*0*
26	*1.9*	*5.2*	*4.4*	*2.6*	*0*
27	*2.4*	*6.7*	*2.6*	*3.2*	*0*
28	*3.3*	*7.3*	*2.8*	*6*	*0*
29	*1.9*	*7.4*	*3.4*	*2.8*	*1*
30	*0.8*	*3.7*	*1.1*	*4.5*	*0*
31	*0.9*	*2.1*	*1*	*4.3*	*0*
32	*2.5*	*6.3*	*1.2*	*3*	*1*
33	*2.3*	*14.3*	*2.3*	*5.2*	*1*
34	*1.2*	*2.2*	*2.2*	*5.4*	*0*
35	*1.7*	*3.9*	*1.7*	*4*	*0*
36	*1.4*	*5.8*	*3.5*	*4.2*	*0*
37	*2.1*	*5.7*	*3*	*5.4*	*0*

For details please see Table (**1b**).

**Table 1b:** Grading OCT score for AMD eyes enrolled in this study.

**ID**	**1**	**2**	**3**	**4**	**5**	**6**	**7**	**8**	**9**	**10**	**11**	**12**	**13**	**14**	**15**	**16**	**17**	**18**	**19**	**20**
**Inner Retinal Surface**																				
Normal foveal structure (0)				*0*				*0*				*0*		*0*		*0*	*0*	*0*		
Flattened (1)					*1*	*1*													*1*	
Elevated (2)	*2*		*2*						*2*	*2*	*2*		*2*		*2*					*2*
Bumpy (3)		*3*					*3*													
**Retinal Structure**																				
Normal(0)	*0*			*0*	*0*			*0*						*0*			*0*	*0*		
Thickened(1)							*1*		*1*	*1*						*1*			*1*	*1*
Cystic changes(2)		*2*				*2*					*2*		*2*		*2*					
Atrophic(3)			*3*									*3*								
**Photoreceptor Layers**																				
Normal(0)					*0*			*0*						*0*						
Elevated(1)	*1*		*1*				*1*													
Bumpy(2)						*2*			*2*	*2*	*2*		*2*				*2*	*2*	*2*	*2*
Atrophic(3)		*3*		*3*								*3*			*3*	*3*				
**Pigment Epithelium**																				
Normal(0)																				
Elevated(1)											*1*									
Bumpy(2)	*2*	*2*	*2*	*2*	*2*	*2*	*2*	*2*	*2*	*2*		*2*	*2*	*2*			*2*	*2*	*2*	*2*
Atrophic(3)															*3*	*3*				
**Score**	**5**	**10**	**8**	**5**	**3**	**7**	**7**	**2**	**7**	**7**	**7**	**8**	**8**	**2**	**10**	**7**	**4**	**4**	**6**	**7**

**Table 2:** shows mean sensitivity and specificity values of self-monitoring tests in AMD (taken from Faes *et al*., 2014) [10].

**Index Test**	**# Studies**	**Mean Sensitivity [%]**	**Mean Specificity [%]**
**Amsler Grid**	9	70.9	93.3
**PHP**	10	85.5	86.9
**M-chart**	2	81.3	100.0
**Macular Computerized Psychophysical Test**	1	93.8	94.1
**PHP Home Device**	2	84.8	85.0
**Modified Amsler Grid**	2	74.3	97.6
**3D-computer-automated Amsler Grid**	1	100.0	100.0
